# Parasitic copepods from Egyptian Red Sea fishes: Bomolochidae Claus, 1875

**DOI:** 10.1007/s11230-015-9618-4

**Published:** 2016-01-20

**Authors:** Hoda Hassan El-Rashidy, Geoffrey Allan Boxshall

**Affiliations:** Department of Oceanography, Faculty of Science, Alexandria University, Moharram Bey, Alexandria, Egypt; Department of Life Sciences, Natural History Museum, Cromwell Road, London, SW 7 5BD UK

## Abstract

Two species of parasitic copepods from the genus *Bomolochus* von Nordmann, 1832 (Cyclopoida: Bomolochidae) are redescribed in detail, based on material collected from the gills of Red Sea fishes. Host material was caught at El-tor, near Sharm El-Sheikh, and in the Gulf of Suez, Egypt. Both sexes of *Bomolochus bellones* Burmeister, 1835 were collected from the gills of a needlefish *Tylosurus choram* (Rüppell) caught in the Gulf of Suez. This is a new host record. The female is well characterised so only the male is described. Adult females of *Bomolochus minus* Lin & Ho, 2005 were obtained from the branchial cavities and gills of mojarra *Gerresoyena* (Forsskål). This species was known only from its original description in Taiwan, and this report constitutes a new host record and a significant range extension. Both parasite species are new records for Egyptian Red Sea waters.

## Introduction

The family Bomolochidae Claus, 1875 currently comprises about 141 species of parasites which commonly inhabit the branchial chamber and gills of their marine fish hosts (Boxshall & Halsey, 2004). The type genus, *Bomolochus* von Nordmann, 1832, is the second largest in the family and the review of Ho & Lin (2009) recognised 20 species as valid. Two species are reported here, *Bomolochus bellones* Burmeister, 1835 and *B. minus* Lin & Ho, 2005.

*Bomolochus bellones* was originally discovered off Helgoland, Germany by Burmeister (1835) as a parasite on *Belone belone* (L.) (as *Esox bellone* L.), and was subsequently reported from north-western European waters and the Mediterranean (see Vervoort, 1962; Kabata, 1979). However, it was Cressey & Collette (1970) who first documented the worldwide range of this parasite: they collected hundreds of specimens of *B.bellones* from the gill chambers and oral valves of 16 species of needlefishes (family Belonidae) collected from numerous localities across the North and South Atlantic, the Indian Ocean, and the North and South Pacific (including off Australia). They were the first to report *B.bellones* from the Red Sea, on *Ablennes hians* (Valenciennes), but they did not find this parasite on *Tylosurus choram* (Rüppell). Burmeister’s original description (Burmeister, 1835) was rather rudimentary, but the adult female of *B. bellones* has subsequently been redescribed by numerous authors including Vervoort (1962), Cressey & Collette (1970), Kabata (1979) and Ho et al. (1983). The female is well characterised. However, the only description available of the male is that of Hartmann (1870) who provided a single illustration of an undissected male in ventral view. In the present work, the male *B. bellones* is described in detail, based on material obtained from *Tylosurus choram* caught in the Gulf of Suez.

The only previous report of *B. minus* is the original description based on material from five different host fishes landed in Taiwan (Lin & Ho, 2005). This species is very closely related to the poorly described species, *B. indicus* Kaliyamurthy, Singh & Singh, 1988 and may even be a junior synonym. The females from Egyptian waters are redescribed here to add to the body of morphological knowledge that will provide the evidence which will allow such questions of possible synonymy to be addressed.

## Materials and methods

Host fish were purchased from local markets and examined for the presence of parasitic copepods. Copepods were removed from the host and preserved in 70% ethanol. The copepods were dissected and mounted in lactophenol as temporary slide preparations and examined on an Olympus microscope. Measurements were made using an ocular micrometer and drawings were made with the aid of drawing tube. Morphological terminology follows Huys & Boxshall (1991). Host names were validated against FishBase (Froese & Pauly, 2015).

**Family Bomolochidae Sumpf, 1871**

***Bomolochus*von Nordmann, 1832**

***Bomolochus bellones*Burmeister, 1835**

Syns *Parabomolochus bellones* (Burmeister, 1835); *Bomolochus ardeolae* Krøyer, 1863; *Artacolax ardeolae* (Krøyer, 1863); *Holobomolochus ardeolae* (Krøyer, 1863); *Bomolochus concinnus* Wilson, 1911; *Parabomolochus concinnus* (Wilson, 1911); *Bomolochus hemirhamphi* Pillai, 1965; *Parabomolochus hemirhamphi* (Pillai, 1965); *Bomolochus hyporhamphi* Yamaguti & Yamasu, 1959; *Parabomolochus hyporhamphi* (Yamaguti & Yamasu, 1959); *Pseudartacolax hyporhamphi* (Yamaguti & Yamasu, 1959); *Bomolochus tumidus* Shiino, 1957; *Parabomolochus tumidus* (Shiino, 1957); *Artacolax tumidus* (Shiino, 1957)

*Host*: *Tylosurus choram* (Rüppell).

*Locality*: Gulf of Suez (Red Sea), Egypt.

*Site on host*: Gills, branchial cavity.

*Material examined*: 25 adult females and 2 males.

### Description (Figs. 1–2)

**Fig. 1:**
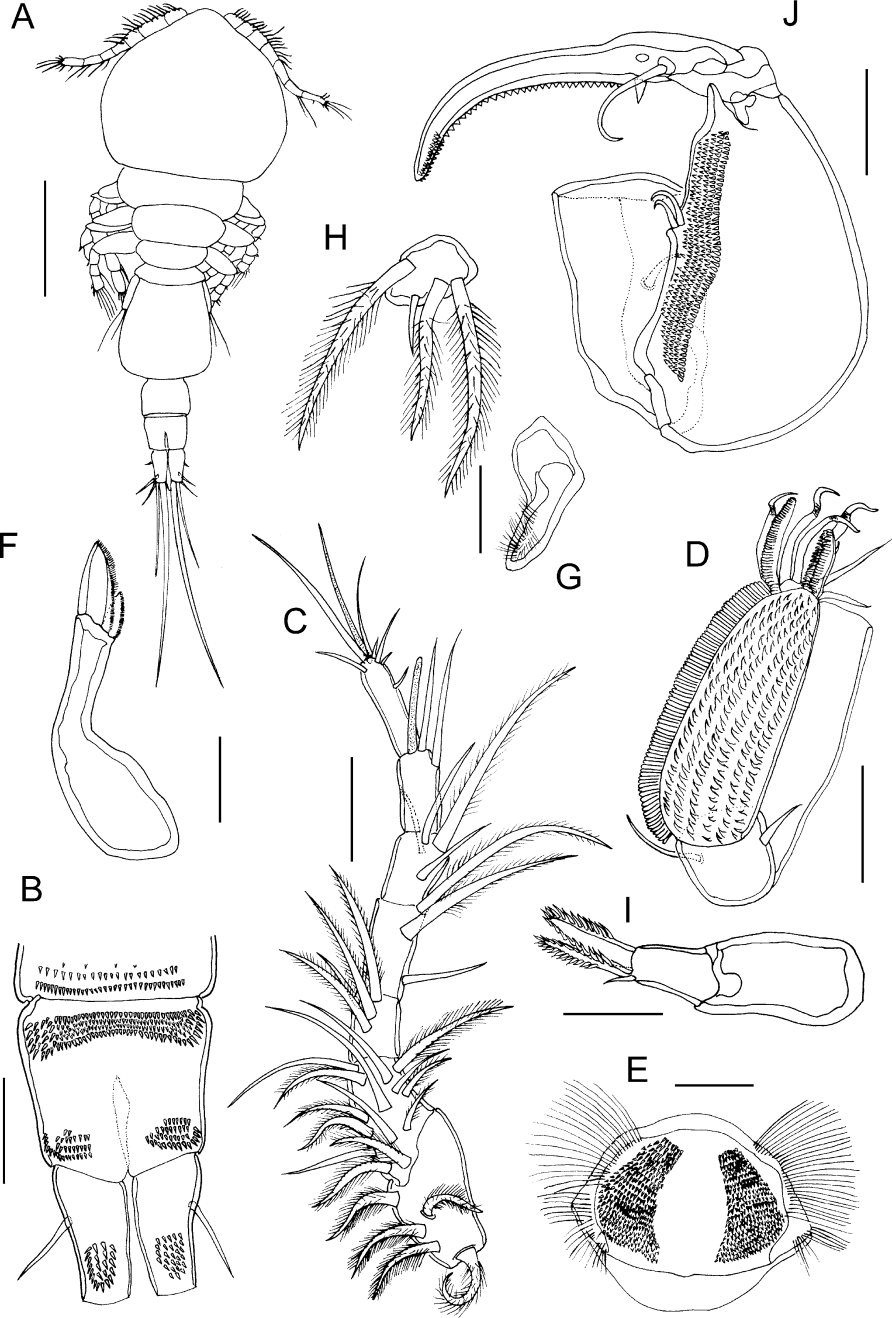
*Bomolochus bellones* Burmeister, 1835. Adult male. A, Habitus, dorsal view; B, Anal somite and caudal rami, ventral view; C, Antennule; D, Antenna; E, Labrum; F, Mandible; G, Paragnath; H, Maxillule; I, Maxilla; J, Maxilliped. *Scale-bars*: A, 0.25 mm; B–D, J, 50 µm; E–I, 25 µm

*Adultmale*. Body cyclopiform (Fig. 1A); 0.87–0.98 (0.93) mm long (based on 2 specimens). Prosome length 0.46–0.54 (0.50) mm, maximum width 0.28–0.35 (0.31) mm; comprising cephalothorax incorporating first pedigerous somite, and free second to fourth pedigerous somites. Urosome (Fig. 1A, B) length 0.37–0.44 (0.41) mm; comprising fifth pedigerous somite, pear-shaped genital somite and 2 free abdominal somites. Ventral surface of first free abdominal somite ornamented posteriorly with rows of spinules. Anal somite deeply incised, ornamented with transverse patch of spinules anteriorly and paired patches posteriorly (Fig. 1B). Caudal rami (Fig. 1B) ornamented with ventral patch of spinules.

**Fig. 2:**
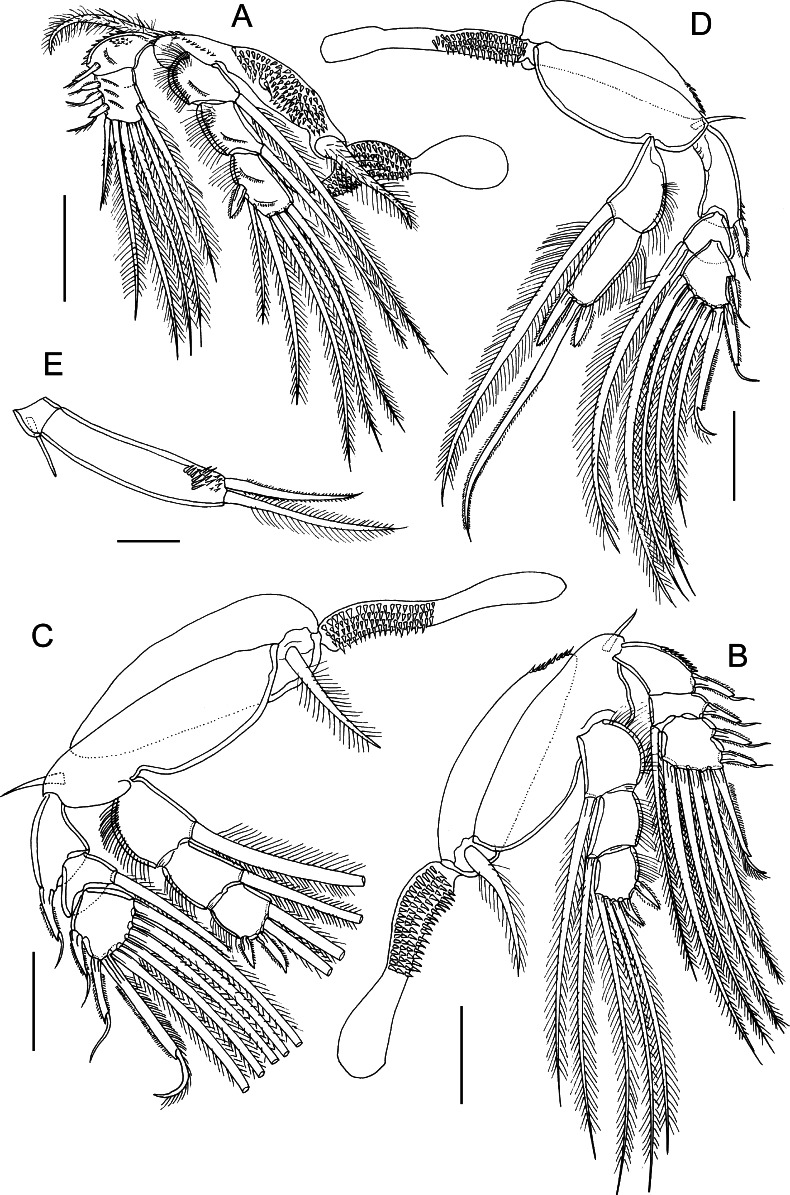
*Bomolochus bellones* Burmeister, 1835. Adult male. A, Leg 1; B, Leg 2; C, Leg 3; D, Leg 4; E, Leg 5. *Scale-bars*: C–D, 100 µm; A–B, 50 µm; E, 25 µm

Antennule (Fig. 1C) 7-segmented; proximal 4 segments only slightly more robust than distal 3 cylindrical segments. First segment with 5 robust pilose setae, none modified; second segment with 14 setae (6 robust pilose setae, 5 naked setae and 3 short plumose setae); third segment with 1 naked seta and 3 robust pilose setae; fourth segment with 1 naked seta and 2 robust pilose setae. Cylindrical distal segments with setal formula 4, 2+1ae and 7+1ae respectively; distal element on fifth segment longer than in female.

Antenna (Fig. 1D) uniramous, 3-segmented; comprising long proximal segment (coxobasis) bearing single long seta, short middle (= first endopodal) segment armed with small naked seta, and highly ornamented apical segment. Apical segment produced into blunt distal process ornamented with marginal row of blunt spinules continuous with row present along margin of apical segment, ventral surface of segment with multiple rows of hooked spinules. Apical segment armed distally with 4 curved claws, 3 naked setae and pectinate process bearing tiny naked seta.

Labrum (Fig. 1E) ornamented with paired patches of spinules on ventral surface and patches of long setules laterally. Mandible (Fig. 1F) bearing 2 unequal blades distally, each spinulate along one margin. Paragnath (Fig. 1G) forming long blunt process fringed distally with long setules. Maxillule (Fig. 1H) forming rounded lobe armed with 1 naked seta and 3 unequal pilose setae. Maxilla (Fig. 1I) 2-segmented; proximal segment (syncoxa) larger, unarmed; second segment (basis) narrowing distally, bearing 2 spinulate apical elements plus small naked seta.

Maxilliped (Fig. 1J) comprising syncoxa armed with naked seta; basis massive, ornamented medially with multiple rows of hooked spinules and armed medially with 2 naked setae; distal claw incorporating endopodal segment, armed with long seta and small hyaline process proximally, inner margin of claw ornamented with row of denticles plus cluster of spinules at tip.

Legs 1 to 4 biramous, with armature as follows:

**Table:** 

	Coxa	Basis	Exopod	Endopod
Leg 1	0-1	1-0	I-0; III, 1,5	0-1; 0-1; I,5
Leg 2	0-1	1-0	I-0; I-1; II,I,4	0-1; 0-1; II,3
Leg 3	0-1	1-0	I-0; 0-1; II,I,5	0-1; 0-1; II,2
Leg 4	0-0	1-0	I-0; 0-1; II,I,4	0-1; I,1,I

Leg 1 (Fig. 2A) rami less flattened and less modified than in female. Protopod ornamented with rows of spinules, armed with plumose outer basal seta and plumose inner seta (not flattened as in female). Interpodal sclerite small, slightly wider than long, ornamented with multiple transverse rows of small spinules (Fig. 2A). Exopod 2-segmented; first segment with one spine at outer distal corner; segments 2 and 3 partially fused ventrally, bearing outer 3 spines, terminal seta, plus 5 inner setae (Fig. 5A). Endopod 3-segmented; all segments ornamented with outer margin setules, first and second segments each with oblique row of small spinules on anterior surface, third segment with 2 rows.

Legs 2 and 3 (Fig. 2B–C) biramous with 3-segmented rami; outer margin spines on exopod segments finely bilaterally spinulate, each spine bearing subterminal flagellum. Ornamentation of long setules present on outer margins of endopodal segments. Coxa of leg 2 with spinules at outer distal angle.

Leg 4 (Fig. 2D) biramous with 3-segmented exopod and 2-segmented endopod; outer margin spines on exopod segments finely bilaterally spinulate, each spine bearing subterminal flagellum. Inner seta on proximal endopodal segment about twice as long as ramus, extending almost to tip of long seta on distal segment; distal endopodal segment with inner apical spine longer than outer; apical seta about 1.5 times longer than ramus. Ornamentation of long setules present on outer margins of endopodal segments. Leg 4 coxa with spinules at outer distal angle.

Leg 5 (Fig. 2E) 2-segmented, protopodal segment small armed with outer seta; free distal segment (exopod) ornamented distally with patches of spinules, armed with 2 unequal terminal setae.

### Remarks

In the 180 years since its discovery, *Bomolochus bellones* has acquired a long synonymy. It became the type-species of the genus *Parabomolochus* erected in 1962 by Vervoort (1962), but just seven years later Vervoort (1969) synonymised *Parabomolochus* with *Bomolochus* and transferred *B. bellones* back under its original binomen. Five other nominal species have been recognised as junior subjective synonyms of *B. bellones*. Cressey (1981) re-examined the type-material of *Bomolochus ardeolae* Krøyer, 1863 from *Platybelone argalus* (LeSueur) (as *Belone ardeola* LeSueur), and concluded that it was a synonym of *B. bellones*. Until Cressey (1981) examined the types, confusion had surrounded the identity of *Bomolochus ardeolae*, exacerbated by the publication of Wilson (1908) who mistakenly identified and described material from *Hypsypops rubicunda* (Girard), caught off California, under the name *B. ardeolae*. The material of Wilson (1908) was subsequently re-identified as *Holobomolochus glyphysodontis* (Krøyer, 1863) by Cressey (1981). As pointed out by Cressey (1981), the genus *Artacolax* Wilson, 1908 was established with *Bomolochus ardeolae* as its type-species, so *Artacolax* is a synonym of *Bomolochus*.

Cressey (1983) also re-examined the type-material of *Bomolochus concinnus* Wilson, 1911, collected from *Strongylura marina* (Walbaum) (as *Tylosurus marinus*) (Wilson, 1911), and considered that it was conspecific with *B. bellones*. Bere (1936) reported *B. nitidus* Wilson, 1911 from *Strongylura timucu* (Walbaum) (as *Strongylura timuca*) caught off the west coast of Florida (USA). Pillai (1967) considered this to be a misidentification, noting strong differences between Bere’s material and the original description of *B. nitidus* given by Wilson (1911). Pillai (1967) suggested that Bere’s material was conspecific with *B. concinnus* Wilson, 1911 which Cressey (1983) had placed in synonymy with *B. bellones*. *Bomolochus nitidus* Wilson, 1911 is a valid species (Ho & Lin, 2009) parasitic on mugilids.

In 1983, Ho et al. (1983) concluded that three Indo-West Pacific species, *B. tumidus* Shiino, 1957, *B. hyporhamphi* Yamaguti & Yamasu, 1959 and *B. hemirhamphi* Pillai, 1965, were all conspecific with *B. bellones*. Accepting all these synonymies adds several other host species, including halfbeaks (family Hemiramphidae) and the saury (family Scomberesocidae), to the known range of fishes utilised by *B. bellones*. Collette (1974) reported *B. bellones* from an additional three species of *Hyporhamphus* (Table 1).

**Table 1:** Known hosts of *Bomolochus bellones* Burmeister, 1835

Host family/ species	Parasite	Reference
Belonidae		
*Ablennes hians* (Valenciennes)	*Parabomolochus bellones* (Burmeister, 1835)	Cressey & Collette (1970)
(as *Tylosurus schismatorhynchus*)	*Bomolochus tumidus* Shiino, 1957	Shiino (1957)
	*Bomolochus hyporhamphi* Yamaguti & Yamasu, 1959	Yamaguti & Yamasu (1959)
*Belone belone* (Linnaeus) (as *B. vulgaris*, *B. rostrata* or *Esox bellones*)	*Bomolochus bellones* Burmeister, 1835	Burmeister (1835); Heller (1865); Hartmann (1870); Brian (1902, 1906); Leigh-Sharpe (1933); Kabata (1979); Vervoort (1962)
	*Parabomolochus bellones* (Burmeister, 1835)	Cressey & Collette (1970)
*Belone svetovidovi* Collette & Parin	*Parabomolochus bellones* (Burmeister, 1835)	Cressey & Collette (1970)
*Platybelone argalus* (LeSueur)	*Parabomolochus bellones* (Burmeister, 1835)	Cressey & Collette (1970)
(as *Belone ardeola*)	*Bomolochus ardeolae* Krøyer, 1863	Krøyer (1863)
*Strongylura anastomella* (Valenciennes)	*Parabomolochus bellones* (Burmeister, 1835)	Cressey & Collette (1970)
*Strongylura incisa* (Valenciennes)	*Parabomolochus bellones* (Burmeister, 1835)	Cressey & Collette (1970)
*Strongylura leiura* (Bleeker)	*Parabomolochus bellones* (Burmeister, 1835)	Cressey & Collette (1970)
*Strongylura marina* (Walbaum)	*Bomolochus concinnus* Wilson C. B., 1911	Wilson (1911)
	*Parabomolochus bellones* (Burmeister, 1835)	Cressey & Collette (1970)
*Strongylura notate* (Poey)	*Parabomolochus bellones* (Burmeister, 1835)	Cressey & Collette (1970)
*Strongylura senegalensis* (Valenciennes)	*Parabomolochus bellones* (Burmeister, 1835)	Cressey & Collette (1970)
*Strongylura strongylura* (van Hasselt)	*Parabomolochus bellones* (Burmeister, 1835)	Cressey & Collette (1970)
*Strongylura timucu* (Walbaum)	*Bomolochus nitidus* Wilson C. B., 1911	Bere (1936)
	*Parabomolochus bellones* (Burmeister, 1835)	Cressey & Collette (1970)
*Strongylura urvillii* (Valenciennes)	*Parabomolochus bellones* (Burmeister, 1835)	Cressey & Collette (1970)
*Tylosurus acus* (Lacépède)	*Parabomolochus bellones* (Burmeister, 1835)	Cressey & Collette (1970)
*Tylosurus choram* (Rüppell)	*Bomolochus bellones* Burmeister, 1835	Present account
*Tylosurus crocodilus* (Pèron & LeSueur)	*Parabomolochus bellones* (Burmeister, 1835)	Cressey & Collette (1970)
*Tylosurus gavialoides* Castlenau (as *Lhotskia gavialoides*)	*Parabomolochus bellones* (Burmeister, 1835)	Cressey & Collette (1970)
Hemiramphidae		
*Hemiramphus far* (Forsskål)	*Bomolochus hemirhamphi* Pillai, 1965	Pillai (1965, 1985)
*Hyporhamphus australis* (Steindachner)	*Bomolochus bellones* Burmeister, 1835	Collette (1974)
*Hyporhamphus melanochir* (Valenciennes)	*Bomolochus bellones* Burmeister, 1835	Collette (1974)
*Hyporhamphus regularis* (Günther)	*Bomolochus bellones* Burmeister, 1835	Collette (1974)
*Hyporhamphus sajori* (Temminck & Schlegel)	*Bomolochus hyporhamphi* Yamaguti & Yamasu, 1959	Yamaguti & Yamasu (1959)
	*Bomolochus bellones* Burmeister, 1835	Ho et al. (1983)
Scomberesocidae		
*Cololabis saira* (Brevoort)	*Bomolochus tumidus* Shiino, 1957	Shiino (1957)

Males of *Bomolochus* are found and reported less frequently than females. Currently males are known for just seven of the 20 valid species of *Bomolochus*: *B. bellones*, *B. ensiculus* (Cressey, in Cressey & Collette, 1970), *B. globiceps* (Vervoort & Ramirez, 1968), *B. megaceros* Heller, 1865, *B. psettobius* (Vervoort, 1962), *B. soleae* Claus, 1864 and *B. xenomelanirisi* Carvalho, 1955. In addition, Vervoort (1969) described an unidentified male as *Bomolochus* sp., although this specimen may well have been immature (Vervoort, 1969). The only description of male *B. bellones* is that of Hartmann (1870) which is supported by a single illustration of an undissected male, providing an inadequate level of detail. The detailed description presented here permits comparison with congeneric males.

Adult males of *Bomolochus* spp. are all smaller than their respective females. Sexual dimorphism is exhibited in urosomal segmentation (the formation of the genital somite and the number of free abdominal somites), in the antennule (the lack of the modified hook-like seta on the proximal segment in the male), the robust subchelate maxilliped of the male, the 2-segmented endopod of leg 4 in the male (3-segmented in the female), and the presence of only two setal elements on the free exopodal segment of leg 5 in the male (compared to four setal elements in the female). The spine and setal formula for the exopods of legs 3 and 4 is also sexually dimorphic: adult males lack an outer spine on the second exopodal segment of both legs, whereas a spine is present in the females. There are additional differences in ornamentation: the male has extensive spinulation on the ventral surface of the first free abdominal and anal somites, on the caudal rami, and on the interpodal sclerites of legs 1 to 4. This enhanced spinular ornamentation presumably assists the male in holding the female during mating.

***Bomolochusminus*Lin & Ho, 2005**

*Host*: *Gerres oyena* (Forsskål).

*Locality*: El-Tor, Egypt, Red Sea.

*Site on host*: *Gill cavity.*

*Material examined*: 4 females.

### Description (Figs. 3–5)

**Fig. 3:**
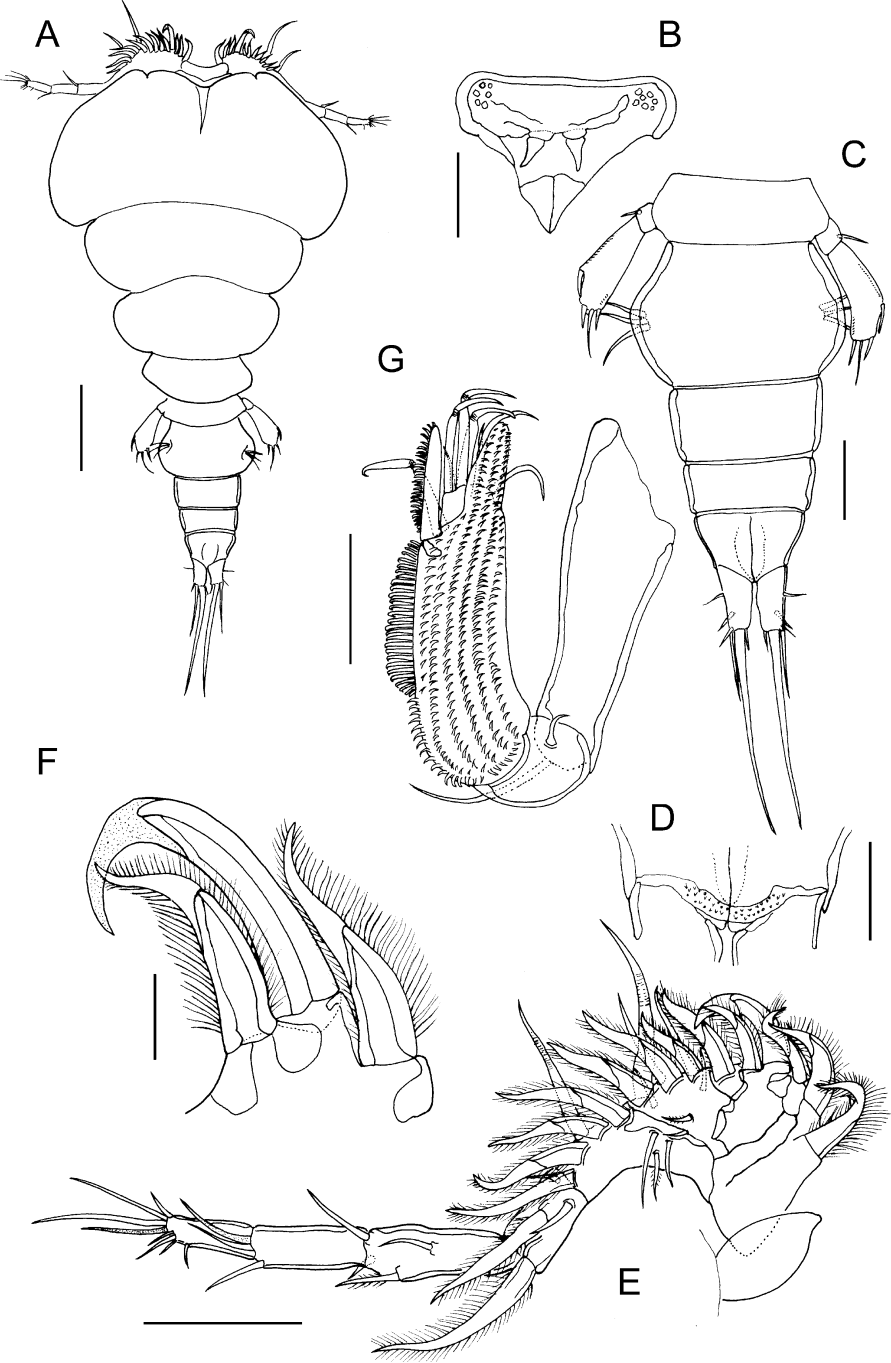
*Bomolochus minus* Lin & Ho, 2005. Adult female. A, Habitus, dorsal view; B, Rostrum, ventral view; C, Urosome; D, Articulation between anal somite and caudal rami; E, Antennule; F, Proximal part of first antennulary segment showing modified fourth seta and adjacent setae; G, Antenna. *Scale-bars*: A, 250 µm; C, E, 100 µm; B, D, G, 50 µm; F, 25 µm

*Adult female.* Body cyclopiform (Fig. 3A); 1.49 to 1.57 (1.53) mm long (based on 4 specimens); prosome length 0.95–1.06 (1.00) mm, maximum width 0.69–0.88 (0.86) mm. Prosome comprising broad cephalothorax and free second to fourth pedigerous somites; third somite not overlapping fourth in dorsal view (Fig. 3A). Cephalothorax bearing pair of acutely pointed tines in rostral area (Fig. 3B). Urosome (Fig. 3C) 0.51–0.60 (0.56) mm long, comprising fifth pedigerous somite, genital double-somite and 3 free abdominal somites. All urosomites wider than long; second free abdominal somite shortest; anal somite deeply incised posteromedially. Surfaces of all urosomites and caudal rami smooth, lacking ornamentation of spinules, but minute denticles present on membrane between anal somite and caudal rami (Fig. 3D). Caudal rami (Fig. 1C) twice as long as wide, bearing single principal seta, plus 5 small setae.

**Fig. 4:**
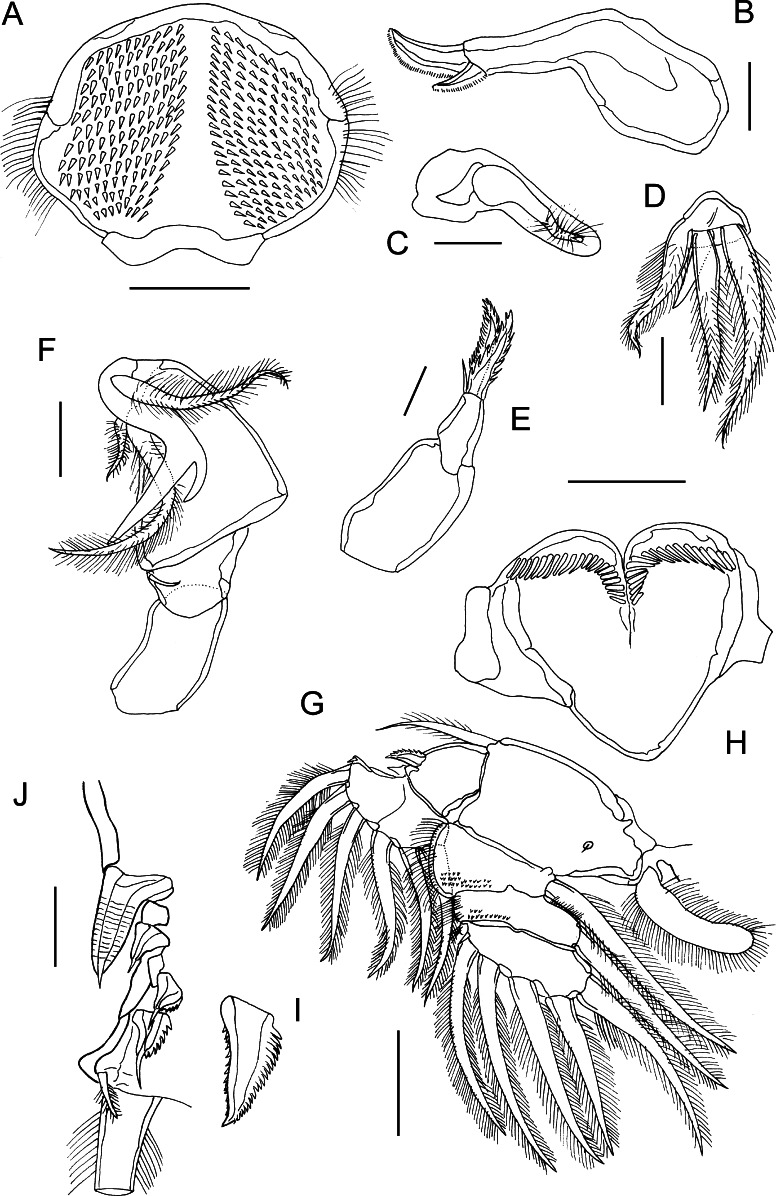
*Bomolochus minus* Lin & Ho, 2005. Adult female. A, Labrum; B, Mandible; C, Paragnath; D, Maxillule; E, Maxilla; F, Maxilliped; G, Leg 1; H, Interpodal sclerite of leg 1; I, Outer spine on exopodal segment 1; J, Outer spines on compound distal exopodal segment. *Scale-bars*: A, H, 50 µm; B–F, I–J, 25 µm; G, 100 µm

**Fig. 5:**
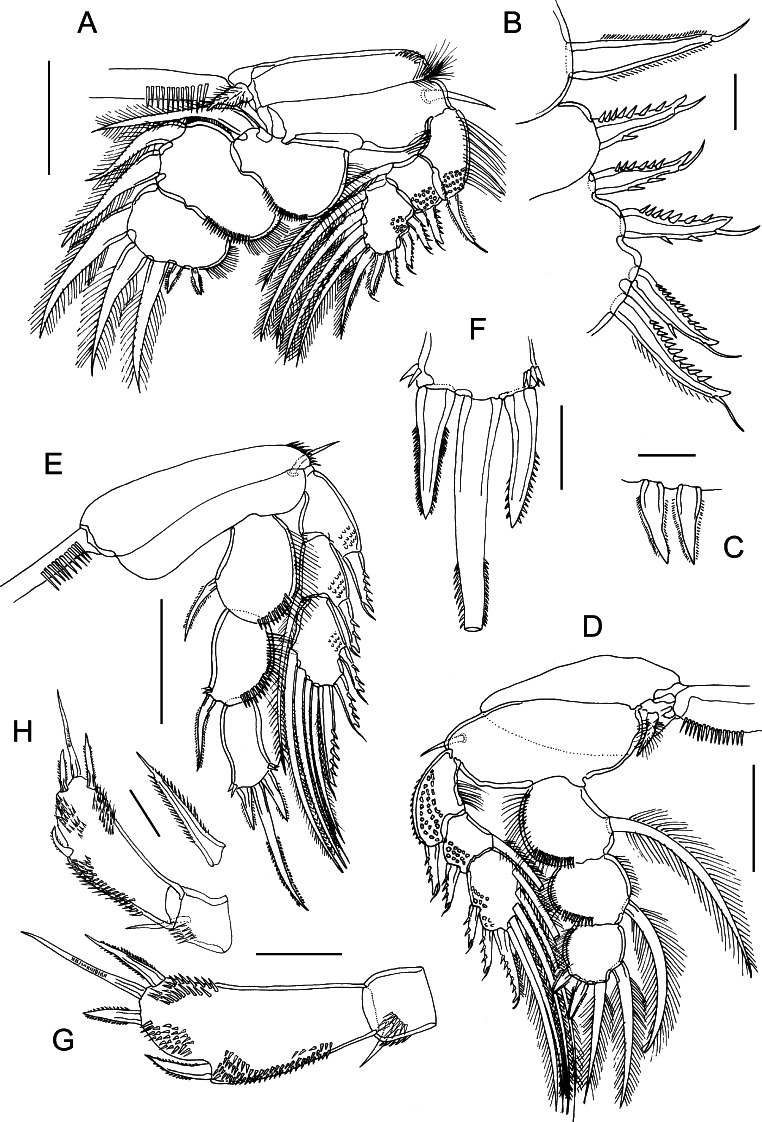
*Bomolochus minus* Lin & Ho, 2005. Adult female. A, Leg 2; B, Outer spines on exopod of leg 2; C, Outer spines on terminal segment of endopod of leg 2; D, Leg 3; E, Leg 4; F, Spines and seta on terminal segment of endopod leg 4; G, Leg 5; H, Leg 5 in another specimen. *Scale-bars*: A, D–E, 100 µm; G, 50 µm; B–C, F, 25 µm

Antennule (Fig. 3E) comprising heavily sclerotised proximal part and slender distal part; proximal part indistinctly 4-segmented, distal part slender, comprising 3 segments. First segment bearing 5 robust pilose setae, fourth seta modified, hook-shaped, only extending slightly beyond tip of adjacent fifth seta; distal non-chitinised, recurved part of fourth seta about 1/3 length of fifth seta (Fig. 1F). Segments 2 and 3 bearing total of 19 setae (8 robust pilose setae, 5 small setae on ventral surface, 6 naked setae of various length on dorsal surface); segment 4 bearing 4 setae (2 robust pilose setae, 1 ventral, 1 dorsal naked seta); setal formula of distal part of antennule: 4, 2 + 1 ae, 7 + 1 ae.

Antenna (Fig. 3G) uniramous, 3-segmented; comprising long proximal segment (coxobasis) bearing single long seta, short middle (= first endopodal) segment armed with small naked seta, and highly ornamented apical segment. Apical segment produced into blunt distal process ornamented with rows of spinules ventrally, continuous with multiple rows over ventral surface of segment. Apical segment armed distally with pectinate process, 4 curved claws and 4 naked setae.

Labrum (Fig. 4A) ornamented with 2 large patches of spinules on ventral surface, and irregular rows of long setules along lateral margins. Mandible (Fig. 4B) tipped with 2 unequal blades, each with single spinulate margin. Paragnath (Fig. 4C) elongate, blunt process fringed distally with long setules. Maxillule (Fig. 4D) lobate, armed with 1 naked and 3 unequal pilose setae. Maxilla (Fig. 4E) 2-segmented; proximal segment (syncoxa) larger, unarmed; second segment (basis) narrowing distally, bearing 2 spinulate apical elements plus small naked seta. Maxilliped (Fig. 4F) comprising syncoxa, armed with distal seta; basis armed with 2 pilose setae; terminal (endopodal) segment forming sigmoid claw provided with short accessory process, and bearing pilose seta proximally.

Legs 1 to 4 biramous, with armature as follows:

**Table:** 

	Coxa	Basis	Exopod	Endopod
Leg 1	0-1	1-0	I-0; IV,1,6	0-1; 0-1; I,5
Leg 2	0-1	1-0	I-0; I-1; III,I,5	0-1; 0-2; II,3
Leg 3	0-1	1-0	I-0; I-1; II,I,5	0-1; 0-1; II,2
Leg 4	0-0	1-0	I-0; I-1; II,I,4	0-1; 0-1; I,1,I

Leg 1 (Fig. 4G) modified with flattened rami: protopod with plumose basal outer seta; inner coxal seta transformed into flattened element with rounded tip, fringed with long setules; interpodal sclerite (Fig. 4H) slightly longer than wide, ornamented with V-shaped rows of long spinules. Exopod indistinctly 2-segmented; first segment with one large spine at outer distal corner, spine with spinule rows bilaterally (Fig. 4I); compound distal segment bearing 4 outer spines (Fig. 4J), 1 small seta at base of terminal plumose seta, and 6 plumose setae. First and second endopodal segments each with inner seta and ornamented with surface spinules and outer margin setules; third segment with 5 plumose setae, and minute spine located proximal to base of outermost seta (Fig. 4G).

Leg 2 (Fig. 5A–C) with 3-segmented rami; coxa with short inner coxal seta and ornamented with patch of setules at outer distal angle; basis with outer basal seta. All exopodal segments ornamented with patches of flattened scale-like spinules; all outer spines provided with subterminal flagellum; outer spine on first exopodal segment bilaterally spinulate, but outer margins of spines on second and third segments denticulate (Fig. 5A, B). Outer and inner margins of first exopodal segment ornamented with long setules. Endopodal segments very broad and flattened; outer margins of first and second segments ornamented with long setules and distal row of spinules, third segment with outer row of setules. Interpodal sclerite ornamented with row of long spinules.

Leg 3 (Fig. 5D) with 3-segmented rami; coxa and basis with short inner coxal seta and outer basal seta, respectively. Coxa lacking ornamentation at outer distal angle. Exopodal segments armed and ornamented as for leg 2. Endopodal segments less broad and less flattened than in leg 2; ornamentation as for leg 2. Interpodal plate ornamented with row of long spinules.

Leg 4 (Fig. 5E) with 3-segmented rami; coxa lacking inner seta and ornamented with patch of spinules at outer distal angle; basis with outer basal seta. All exopodal segments ornamented with patches of flattened scale-like spinules; all outer spines denticulate and provided with subterminal flagellum (Fig. 5E). Outer margins of first and second endopodal segments ornamented with long setules and distal row of spinules. Inner seta on first endopodal segment short, extending nearly to middle of second segment. Inner seta on second endopodal segment extending to about 75% length of third segment; small spinules present at base of seta. Third segment with spinules present adjacent to base of outer and inner apical spines. Interpodal sclerite ornamented with row of long spinules.

Leg 5 (Fig. 5G) 2-segmented; protopodal segment small, ornamented with patch of spinules and armed with outer seta; free segment (exopod) armed with subterminal spine extending almost to end of segment, but not extending beyond distal margin (Fig. 5G, H), outer and inner terminal spines, plus naked seta in middle of distal margin; inner distal spine longer than outer (Fig. 5I). Exopod ornamented with patch of spinules extending along outer lateral margin, plus 2 distal patches.

Leg 6 (Fig. 3A) represented by 3 short setae located in egg sac attachment area on genital double-somite.

*Male*: unknown.

### Remarks

Only four nominal species of *Bomolochus* share the unusual scale-like ornamentation on the exopods of swimming legs 2 to 4, namely, *B. decapteri* Yamaguti, 1936, *B. indicus* Kaliyamurthy, Singh & Singh, 1988, *B. minus* and *B. unicirrus* Brian, 1902. The adult females of two of these species, *B. decapteri* and *B. unicirrus*, are characterised by the possession of patches of spinules on the ventral surface of both the anal somite and the caudal rami (cf. Yamaguti, 1936: figure 50; Ho & Rokicki, 1987: figure 1c). *Bomolochusminus* lacks such patches: indeed, the specific epithet “*minus*” specifically alludes to the absence of this ornamentation (Lin & Ho, 2005). Kaliyamurthy et al. (1988) do not mention any such patches of ornamentation on the anal somite or caudal rami, and while the overall quality of their description of *B*. *indicus* is low, it does mention the scale-like ornamentation (referred to as “pustules”) on the exopods of legs 2 to 4, indicating that the authors were paying attention to such fine details. So we presume these spinule patches are absent in *B. indicus* also.

*Bomolochus indicus* and *B. minus* are morphologically very similar, the body length given for the former (1.6 mm) lies within the range (1.52 to 2.00 mm) given for the latter, and both occur on the same host, *Gerresfilamentosus* Cuvier (see Kaliyamurthy et al., 1988; Lin & Ho, 2005). The former also occurred on a second gerreid, *Gerres limbatus* Cuvier (as *Gerres lucidus*), while the latter also occurred on four sciaenids, *Johnius belengerii* (Cuvier), *J. amblycephalus* (Bleeker), *Pennahia pawak* (Lin), and *Chrysochir aureus* (Richardson). Despite sharing a common suite of characters and co-occurring on the gerreid host, *G. filamentosus*, these two species were not compared by Lin & Ho (2005) when they established their new species from Taiwan.

The key to species of *Bomolochus* created by Ho & Lin (2009) separates *B. indicus* and *B. minus* on the basis of the length of the modified, hook-like fourth seta on the proximal segment of the antennule of the female relative to the length of the adjacent fifth seta. In *B. indicus* the fourth seta protrudes well beyond the tip of the fifth seta, whilst in *B. minus* it does not. The interpretation of this character can be difficult as relative lengths can appear to vary according to the angle of observation: for example, in Lin & Ho’s description (Lin & Ho, 2005) the dorsal view of the adult female of *B. minus* shows the fourth seta as markedly longer than the fifth, but in the ventral view of the antennule, they appear similar in length. In the Egyptian material the fourth seta is somewhat intermediate in length: in Fig. 3F the enlargement of the third, fourth and fifth setae, the hook-like fourth seta is shown extending a small distance beyond the tip of the fifth seta. On the basis of this character we identify the Egyptian material from *Gerres oyena* as *B. minus*, but we recommend that the *B. indicus* is fully redescribed to modern standards because we consider it possible that these two *Bomolochus* species are conspecific.
